# One-Step Automatic Radiosynthesis and Evaluation of [^18^F]TM-30089 as GPR44 Radiotracer

**DOI:** 10.3390/ph16101480

**Published:** 2023-10-17

**Authors:** Jiangling Peng, Wei Tang, Jeffrey Rawson, Lynn Miao, Nelson Gonzalez, Runkai Yin, Jiaqi Chen, Melinda Ji, Zhixuan Li, Anna Gao, Andy Z. Wu, John E. Shively, Fouad Kandeel, Junfeng Li

**Affiliations:** 1 Department of Translational Research & Cellular Therapeutics, Arthur Riggs Diabetes and Metabolism Research Institute, Beckman Research Institute of the City of Hope, Duarte, CA 91010, USA; 2 Department of Immunology & Theranostics, Arthur Riggs Diabetes and Metabolism Research Institute, Beckman Research Institute of the City of Hope, Duarte, CA 91010, USA

**Keywords:** G protein-coupled receptor 44 (GPR44), ^18^F-labeling, chemoattractant receptor-homologous molecule expressed on T-helper type 2 cells (CRTH2), prostaglandin D_2_ receptor 2 (DP2), prostaglandin D_2_ (PGD_2_), inflammation, automatic model, diabetes, cancer, human islets

## Abstract

Recently, a G-protein coupled receptor 44 (GPR44) was discovered to play a significant role in the process of inflammation-related diseases, including cancer and diabetes. However, the precise role of GPR44 has yet to be fully elucidated. Currently, there is a strong and urgent need for the development of GPR44 radiotracers as a non-invasive methodology to explore the exact mechanism of GPR44 on inflammation-related diseases and monitor the progress of therapy. TM-30089 is a potent GPR44 antagonist that exhibits a high specificity and selectivity for GPR44. Its structure contains a fluorine nuclide, which could potentially be replaced with ^18^F. In the present study, we successfully took a highly effective synthesis strategy that pretreated the unprotected carboxylic acid group of the precursor and developed a feasible one-step automatic radiosynthesis strategy for [^18^F]TM-30089 with a high radiochemical purity and a good radiochemical yield. We further evaluated this radiotracer using mice models implanted with 1.1 B4 cell lines (GPR44-enriched cell lines) and human islets (high GPR44 expression), respectively. The results revealed the persistent and specific uptake of [^18^F]TM-30089 in GPR44 region, indicating that [^18^F]TM-30089 is a promising candidate for targeting GPR44. Further evaluation is ongoing.

## 1. Introduction

G protein-coupled receptor 44 (GPR44), alternatively known as the chemoattractant receptor-homologous molecule expressed on T-helper type 2 cells (CRTH2) or prostaglandin D_2_ receptor 2 (DP2) [1,2], was first identified by Marchese et al. in 1999 [3]. Structurally, it is composed of seven transmembrane alpha helices and is closely related to chemoattractant receptors [4]. It is one of two G-protein-coupled receptors recognized for their high-affinity binding to prostaglandin D_2_ (PGD_2_), which is known to be involved in a vast range of physiological and pathophysiological processes [5]. The PGD_2_-GPR44 pathway is implicated in a variety of diseases, including those of the central nervous system [6], urinary tract [7], gastrointestinal tract [8], respiratory system [9], integumentary system [10], osseous and chondral tissues [11], and a range of cancers [12].

Recently, GPR44 is becoming a novel target to aid the further exploration of the relationship between inflammation and the biologic behavior of tumor/diabetes. This exploration holds the potential to uncover innovative therapeutic approaches. Inflammation is commonly believed to be an immune system defense to control tissue damage, yet under inappropriate conditions with excessive levels, inflammation can also cause tissue damage. In extreme conditions, chronic inflammation may promote tumor progression, which is often referred to as inflammation-induced cancer [13]. Given its established role as an inflammation marker, GPR44 is being investigated for its relevance in understanding the biological behavior of the tumor, including breast cancer [14], colorectal cancer [15], hepatocellular carcinoma [16], gastric cancer [17], leukemia [18], lung cancer [19], and myeloma [20]. However, the precise mechanism underlying the role of GPR44 in inflammation-induced cancer remains unknown, highlighting the need for additional studies to elucidate the exact mechanisms involved. Regarding its relationship with diabetes, Abadpour et al. reported that GPR44 inhibition via selective antagonists could be beneficial for the preservation of islet function under inflammatory and hyperglycemic conditions [21]. However, the clinical trial of AZD1981, which was an orally administered GPR44 antagonist, did not show a significant improvement in insulin secretion [22]. As a result of clinical inconsistencies, as well as the still uncertain relationship between GPR44-modulated insulin secretion and inflammation in vivo, more investigation into the exact mechanisms of GPR44 is imperative [23]. Additionally, GPR44 also shows a paradoxical activity level on type 1 diabetes (T1D) and type 2 diabetes (T2D) [24,25]. The exact mechanism of GPR44 in diabetes with inflammation remains a mystery.

Positron emission tomography (PET), a non-invasive in vivo imaging technique, has emerged as a profoundly sensitive and versatile medical imaging tool. It employs target-specific radiotracers to visualize and quantify molecular as well as functional processes within an organism [26]. GPR44 PET imaging holds the potential to offer invaluable insights into the in vivo function, activity, and localization of GPR44. It could facilitate the deeper exploration of the precise mechanisms underlying therapeutic interventions. Currently, there is a strong and urgent need for the development of suitable GPR44 PET imaging tracers that can be used for these purposes. Nevertheless, there were only three GPR44 PET tracers reported. Two of the three tracers were ^11^C-labeled, namely [^11^C]AZ12204657 and [^11^C]MK-7246 [27,28]. However, ^11^C tracers are significantly limited by the extremely short half-life of ^11^C (20 min), hindering administration to multiple patients, delivery to remote sites, and implementation in pre-clinical validation assays. Manufacturing ^11^C tracers relies on an on-site cyclotron, introducing additional challenges. Compared to ^11^C-labeled tracers, ^18^F-labeled tracers are preferable due to their prolonged half-life (110 min), enabling up to 10 h of PET imaging, mass production, and bulk distribution. Additionally, fluorine-18 demonstrates high positron emission (97%) and low maximum positron energy (0.635 MeV). Despite the various advantages of ^18^F-labeled tracers, there is only one fluorine-18 tracer, named [^18^F]MK-7246, that has been reported [29], and its investigation is still ongoing. The development of the ^18^F GPR44 radiotracer is currently in its initial stages. Consequently, there is a necessity to develop new and potential ^18^F-labeled candidates for various objectives in preclinical or clinical uses.

**Figure 1:**
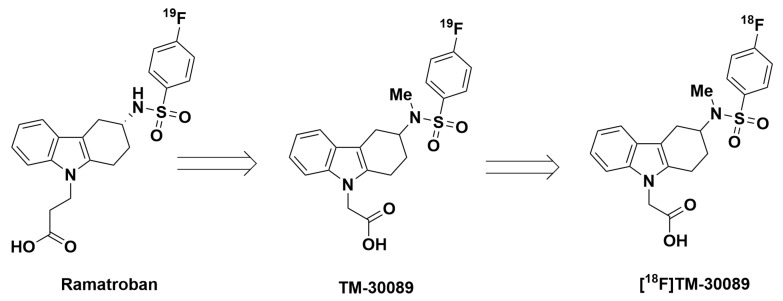
The chemical structures of ramatroban and [^19^F]/[^18^F]TM-30089.

Our research aimed to explore, evaluate, and develop new fluorine-18 GPR44 tracers, which could provide more options for potential tracers in in vivo PET GPR44 imaging. To improve the efficiency of developing new radiotracers, we employed known potent GPR44-targeting antagonists containing a fluorine nuclide, which potentially could be isotopically replaced by an ^18^F. The upside of such an approach was those potent ligands (nonradioactive) were thoroughly characterized (affinity, lipophilicity, protein binding, etc.). Moreover, by the radioisotopic replacement, they ideally preserved their pharmacokinetic properties. In the scope of our present study, TM-30089 ({3-[(4-fluoro-benzenesulfonyl)methyl-amino]-1,2,3,4-tetrahydro-carbazol-9-yl}acetic acid) containing a fluorine nuclide, was selected from an initial pool [30,31]. TM-30089 as a GPR44 antagonist (Figure 1), exhibited the optimal measures of high affinity, specificity, lipophilicity, intrinsic clearance, and bioavailability levels [32]. The potent binding and negligible off-target activity of TM-30089 made it an especially ideal candidate for developing a GRP44 radiotracer [30]. A challenge arose from the presence of a carboxylic acid group in a number of GPR44 antagonists containing a fluorine nuclide (such as TM-30089), culminating in notably diminished radiochemical yield. This issue substantially impeded the applicability in pre-clinical and clinical contexts. In the present study, we explored the development of a novel and feasible one-step automated synthesis methodology, involving the pretreatment of an unprotected carboxylic acid group of the precursor, which had effectively surmounted this issue. This breakthrough stood as a crucial and compelling advancement poised to facilitate the development of GPR44 ^18^F-labeled radiotracers. [^18^F]TM-30089 underwent systematic evaluation in both mouse models implanted with 1.1 B4 (GPR44-enriched tumor cell line) and transplanted human islets (GPR44 high expression), demonstrating its potential for specific GPR44 targeting.

## 2. Results and Discussion

### 2.1. Efficient Identification of Promising ^18^F-Labeled GPR44 Radioligands (Non-Radioactivity Synthesis Section)

There existed a number of GPR44 ligands containing fluorine groups that could serve as a source for the development of potentially ^18^F-labeled GPR44 radiotracers [30,31]. ramatroban (Figure 1), also known as BAY-u 3405, was first described as an antagonist of the thromboxane receptor (TP) and was later shown to selectively bind as an antagonist to GPR44 as well [33]. However, it exhibited approximately 16 times less selectivity for GPR44 than for TP. TM-30089 was a modified ramatroban analog synthesized through the methylation of the nitrogen atom of the sulfonamide group and the replacement of the propanoic acid with an acetic acid. The structural alterations contributed to its strong potency and specificity for GPR44, with a low Ki value of 0.60 nM, and negligible off-target binding to TP and DP1. TM-30089 presented an ideal candidate for the development of the ^18^F labeled GPR44 tracer due to its fluorine-containing structure, which allowed for the minor structural modifications preserve the maximum pharmacokinetic properties, particularly high specificity for GPR44.

### 2.2. Establishment of Murine Models for the Evaluation of GPR44 Radioligands

#### 2.2.1. NOD/SCID Mouse Model with a 1.1 B4 Tumor Cell Line

**Figure 2:**
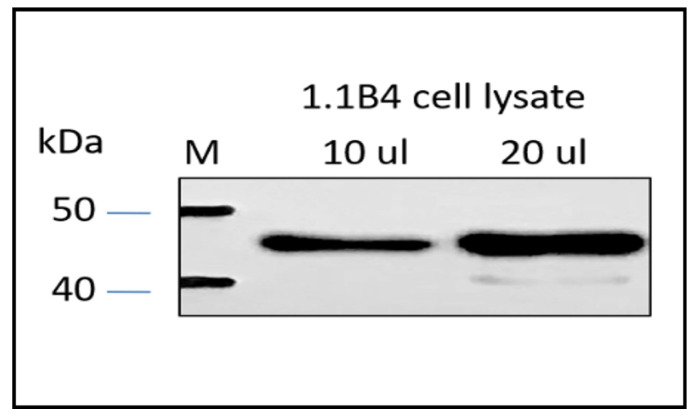
GPR44 expression in human 1.1 B4 cell line via Western blot analysis.

Although GPR44 has high expression in pigs, non-human primates, and humans, only low/no expression is found in mice and rats. This disparity poses a significant barrier in the evaluation of GPR44 radioligands in mouse models. Therefore, it is necessary to establish a viable murine model that accounts for this issue. We hypothesized that the implantation of a human beta cell-like 1.1 B4 tumor cell line, a hybrid line derived from the fusion of a human pancreatic islet culture with a human pancreatic ductal carcinoma cell line (PANC-1), could resolve this issue. Our Western blot analysis demonstrated the expression of GPR44 in the 1.1 B4 cell line (Figure 2). Therefore, in the present study, 1.1 B4 cells were implanted into NOD/SCID mice to generate GPR44-expressing mouse models that could enable an effective evaluation of [^18^F]TM-30089. In this specific murine model, the tissue uniquely targeted by the GPR44 radio-probes is the 1.1 B4-positive tumor.

#### 2.2.2. NOD/SCID Mouse Model Transplanted with Human Islets

**Figure 3:**
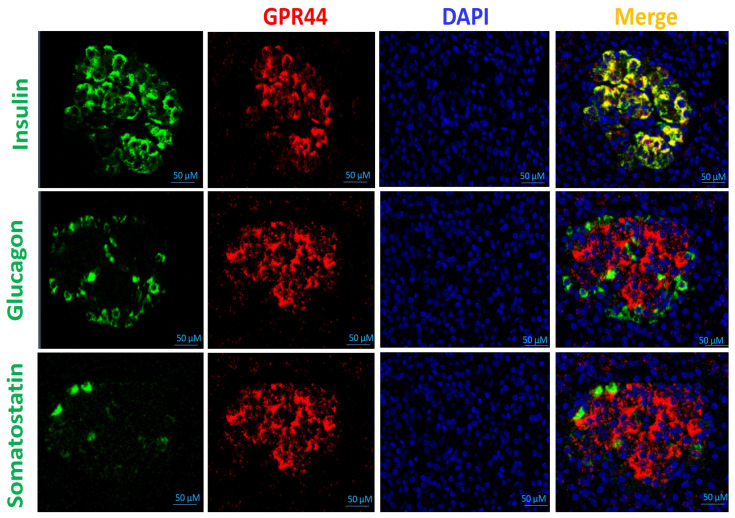
GPR44 expression colocalized with beta cells in human islet. The formalin-fixed, paraffin-embedded tissue sections of adult human pancreases were examined via double and immunofluorescence staining for the GPR44 antibody (red), together with islet major hormones, insulin (green), glucagon (green), and somatostatin (green). DNA was stained blue with DAPI. Data were verified in human pancreases from three to four independently deceased donors.

GPR44 expression in human islet cells was identified via a proteomic screen using the Human Protein Atlas in 2012 [24]. In our study (Figure 3), the tissue sections of the adult human pancreases were examined using double immunofluorescence staining for GPR44 antibody, together with islet major hormones, insulin, glucagon, and somatostatin. The results showed high GPR44 expression in beta cells, where it exhibited strong co-localization with insulin, and a negligible expression in alpha cells and delta cells, as evidenced by minimal co-localization with glucagon or somatostatin. Uppsala University’s team reported similar results, further validating this [23]. Our team routinely performs human islet transplantation in murine renal capsules. This mouse model with human islets were used only for the final evaluation of [^18^F]TM-30089 due to the scarcity of a human islet sources. Only transplanted human islets were targeted by the GPR44 radio-probes.

### 2.3. Radiosynthesis of [^18^F]TM-30089

The analysis of the structure of TM-30089 revealed the presence of an electron-withdrawing sulfonyl group located para-position to the ^19^F nuclide on the phenyl ring. Therefore, nucleophilic aromatic substitution (S_N_Ar) was likely the most viable fluorination method for ^18^F incorporation. Regarding the precursor, the most common and efficient leaving groups for traditional no-carrier-added nucleophilic aromatic substitution were trimethylammonium salt and nitro groups. However, choosing a nitro group as a leaving group required a high reaction temperature (140–150 °C), which could potentially impact product stability and increase the production of unwanted byproducts, causing low radiochemical yield and subsequently limiting future clinical applications. For these reasons, our study utilized trimethylammonium salt as the leaving group, requiring a milder reaction temperature of only 95 °C (see the below section for details). There was a total of seven steps to successfully obtain the precursor (JL01-1) from commercially available starting material.

**Scheme 1:**
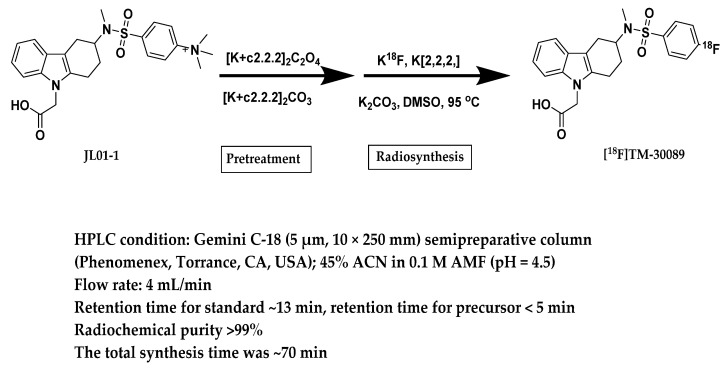
Radiosynthesis of [^18^F]TM-30089 with high radiochemical purity.

In our previous summary, we found that one carboxyl acid group was present in a number of GPR44 antagonists containing a fluorine nuclide. The existence of a carboxyl group within targeted molecules poses a major challenge to direct ^18^F fluorination due to undesirable effects such as lowered radiochemical yields and even an inability to direct nucleophilic radiofluorination. Thus, ^18^F fluorination is typically restricted to application on protected precursors. However, such ^18^F products are labeled with fluorine-18 by laborious, complex multi-step reactions that include the deprotection of these functional groups post-radiofluorination, resulting in low radiochemical yield and a prolonged amount of time. Given the short half-life of ^18^F (approximately 110 min), implementing fast and simple synthesis strategies is highly required to achieve manufacturing standards. Therefore, we conducted an efficient synthesis strategy of the ^18^F-tracer, one that not only enhanced availability but also enabled automation. In our study, we conducted the pretreatment of the carboxylate group of the precursor in the form of a salt with cationic chelates (cryptates, [K^+^c2.2.2]_2_C_2_O_4_, and [K^+^c2.2.2]_2_CO_3_), which was able to effectively avoid the mentioned drawback and attain the synthesis goal (Scheme 1).

The labeling procedure was performed using a Synthra RNplus radio-synthesis module, including one semi-preparative HPLC system (Scheme 1). The precursor of JL01-1 was converted to [^18^F]TM-30089 at 95 °C for 10 min through a one-step labeling reaction. The crude product [^18^F]TM-30089 was purified using the semi-preparative HPLC system in ~13 min with a radiochemical yield of 20–26%. In QC analysis, product [^18^F]TM-30089 was validated, and it had a high radiochemical purity (>99%), as seen using the analytical HPLC system.

### 2.4. Biodistribution

**Figure 4:**
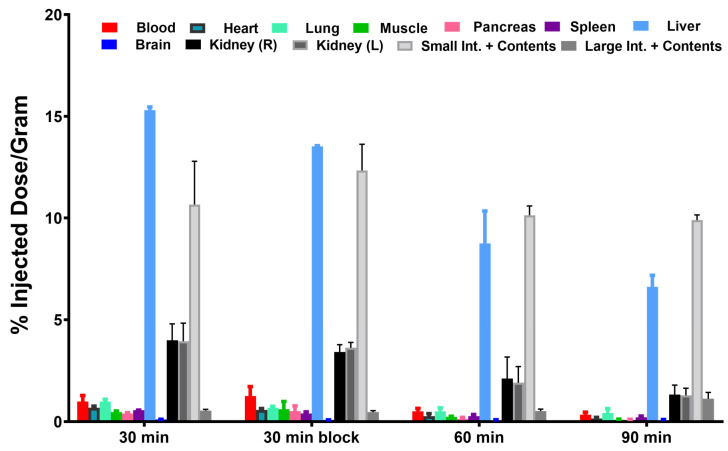
Biodistribution of health NOD/SCID mice at 30, 60-, and 90-min post-injection. Cold TM-30089 as blocking agent (1 mg/kg) was given as treatment at 30 min post-injection.

To obtain the real-time measurements of [^18^F]TM-30089 uptake at various time points, organ/tissue biodistributions in healthy control NOD/SCID mice were performed at 30 min, 60 min, and 90 min post-injection (p.i.), respectively (Figure 4). The result indicated that uptakes in liver and small intestine were highest at all time points (liver: 15.31 ± 0.16% ID/g at 30 min p.i., 8.76 ± 1.60% ID/g at 60 min p.i., and 6.62 ± 0.57% ID/g at 90 min p.i.; small intestine: 10.67 ± 2.13% ID/g at 30 min p.i., 10.14 ± 0.47% ID/g at 60 min p.i., and 9.90 ± 0.26% ID/g at 90 min p.i.). The [^18^F]TM-30089 uptake result was similar to the uptakes of [^18^F]MK-7246 in the liver and small intestine, suggesting that uptakes in these tissues were likely related to excretion [29]. The uptake of the tracer in the kidney was ~4.00 ± 0.80% ID/g at 30 min p.i, 2.11 ± 1.06% ID/g at 60 min p.i., and 1.33 ± 0.47% ID/g at 90 min p.i. The kidney displayed the fast uptake of [^18^F]TM-30089 at 30 min p.i followed by a rapid washout from 60 min to 90 min p.i. Other organs such as the heart, lung, pancreas, and spleen exhibited low uptakes. Low tracer uptake levels were common in the majority of organs, excluding excretion organs such as the liver. In the blocking agent study, we did not find any specifically targeted organs/tissues; therefore, results confirmed the low expression of GPR44 in the murine model as expected. Of the time points, 30 min post-injection was chosen to evaluate the next specific GPR44 mouse models. The further evaluation of biodistribution at 60 min and 90 min was not deemed necessary, since we did not find that the results at those time periods differed significantly from the trends observed in this primary testing group.

**Figure 5:**
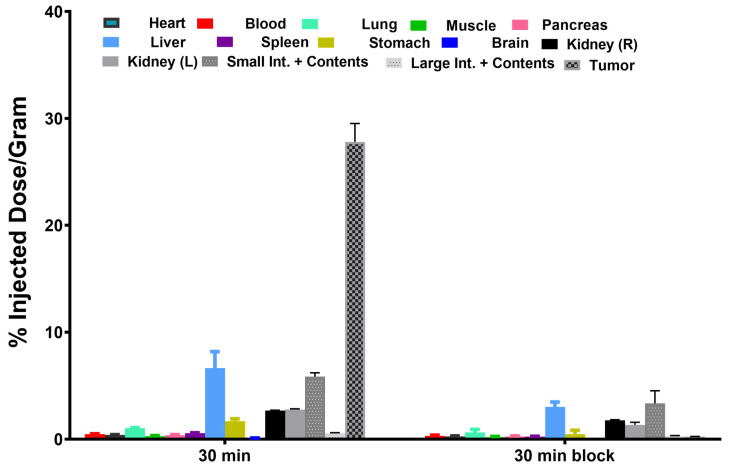
Biodistribution of NOD/SCID mice implanted with 1.1 B4 cells at 30 min after the administration of radiolabel (left graph). Tumor displayed the highest uptake. TM-30089 was given as a blocking agent (1 mg/kg), and organs were collected at 30 min post-injection (right graph). These were significantly blocked.

The biodistribution of [^18^F]TM-30089 was additionally evaluated in NOD/SCID mice with implanted 1.1 B4 cells both with and without the blocking agent. The mice were first anesthetized with 2–4% isoflurane in oxygen before an intravenous injection via the tail vein of ~3.70 MBq (~100 µCi) of [^18^F]TM-30089 was given and were then euthanized at the 30 min point. The uptake values of [^18^F]TM-30089 for the tumors dramatically decreased from 27.81 ± 1.71% ID/g to 0.24 ± 0.02% ID/g in animals injected with cold TM30089 (1 mg/kg), confirming tracer specificity (Figure 5). The uptake in other organs/tissues was consistent with the above biodistribution study in the normal control. Additionally, as expected, the uptake values of the pancreas in both the non-blocked group and the blocked group were low, and the pancreas was not blocked (0.38 ± 0.04% ID/g for non-blocked group and 0.26 ± 0.05% ID/g for blocked group). This observation is in accordance with a known low level of expression of GPR44 in the murine pancreas.

**Figure 6:**
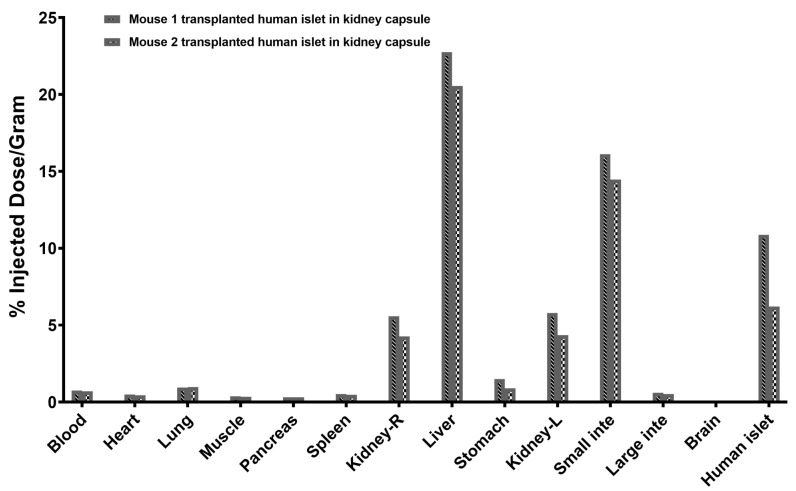
Biodistribution of NOD/SCID mice with transplanted human islets in kidney capsules at 30 min after the administration of the radiolabel. The results indicate the high specific binding to the human islet, in which GPR44 has high expression.

Human islets exhibited the high expression of GPR44. Thus, a mouse model with transplanted 500 IEQ human islets was used for the final evaluation of [^18^F]TM-30089 for a specific binding. A procedure similar to that of the above murine biodistribution was performed in this biodistribution study. Mice were euthanized at 30 min post-injection, and the organs/tissues of interest were collected. Human islets transplanted into a kidney capsule exhibited a notably high uptake (10.87% ID/g and 6.21% ID/g), which demonstrated that [^18^F]TM-30089 had a specific binding in the GPR44-enriched human islets (Figure 6). There was no difference in tracer uptake of other organs/tissues between the human islet-transplanted model and the biodistribution results above.

### 2.5. Feasibility and Acceptability of Screening Strategy

Given the initial stage of developing ^18^F-labeled GPR44 ligands, there exists a vital need to develop new potential candidates to serve various objectives in preclinical or clinical uses. However, the processes of evaluation, screening, and identification of “cold” candidates are crucial steps in the development of PET radiotracers, generally requiring a significant amount of time and resources. To expedite developing the ^18^F-labeled GPR44 ligand, we believed it would be strategically important to use the existing potent analogues that already contain a fluorine-19 nuclide, which could potentially be isotopically replaced by fluorine-18. These fluorine-18 probes do not exhibit the significant modification of pharmacokinetic or other properties, and this strategy has played an important role in the identification of ^18^F-radioligands.

Both [^18^F]TM30089 and previously reported [^18^F]MK-7246 displayed high specific GPR44 binding, where both radiotracers used the above similar screening strategy to select a suitable “cold” ligand during the development of the GPR44 radio-probes. This strategy has been validated as a feasible and successful approach. Based on this efficient and effective screening method, we summarized and recommended a series of ^19^F GPR44 ligands as a potential pool for future PET radiotracer development in our previous review paper [30,31]. However, we addressed a significant concern in the previous section: the presence of one carboxyl acid group in a number of these GPR44 analogues, which could cause diminished radiochemical yields and even indirect nucleophilic radiofluorination (such as [^18^F]TM30089 and [^18^F]MK-7246). In our present study, we successfully overcame this issue, achieving the product in one step with an improved yield. This method could provide sufficient on-demand radiotracer dosages for pre-clinical and even future clinical uses. This feasible one-step synthesis approach has the potential to significantly propel the progression of ^18^F-labeled GPR44 radiotracer development.

Taken together, our results demonstrate that [^18^F]TM-30089 exhibits high specific binding to GPR44, and thus might possess significant potential as an appropriate and efficacious candidate.

## 3. Materials and Methods

### 3.1. Cells and Animals

The hybrid cell line 1.1 B4 (GPR44-positive) was maintained under standard conditions. The 1.1 B4 tumor cells were grown in culture until a sufficient number of cells were available.

Human pancreatic islets were provided by the Southern California Islet Cell Resources Center from human pancreases of healthy adult donors with the proper consent for research use and approval by the Institutional Review Board of the City of Hope.

Male NOD/SCID mice (the City of Hope Animal Resource Center) served as recipients of either 1.1 B4 cells or human islets. This study was carried out in strict accordance with the recommendations of the Guide for the Care and Use of Laboratory Animals of the National Institutes of Health (protocols #15035 and #98001, approved by the Institutional Animal Care and Use Committee of the City of Hope).

#### 3.1.1. Xenograft Tumor Model

NOD/SCID mice (*n* = 24) were subcutaneously injected with 1.1 B4 cells above their shoulders. Tumors were established 17–21 d after injection.

#### 3.1.2. Islet Transplantation

Human islets with diameters of less than 250 µm were used for transplantation. A total of 500 IEQ human islets were transplanted into the renal capsule of NOD/SCID mice (*n* = 5) using a well-established standard procedure [34,35].

### 3.2. Western Blot Analysis

Approximately 10 µg of the denatured protein of 1.1 B4 cells was separated via 10% SDS–polyacrylamide gel electrophoresis and then transferred onto a PVDF (polyvinylidene fluoride) membrane. The membrane was blocked with 5% (*w*/*v*) nonfat milk for 1h at room temperature, and then incubated with the primary antibody anti-CRTH2(1:1000, Abcam, ab190506) overnight at 4 °C, followed by the secondary antibody anti-rabbit IgG (H&L) (1:10,000, Abcam, ab205718) at room temperature for 2 h. The bands were detected using the super-enhanced chemiluminescence (ECL) detection reagent (Thermo Scientific #34579, Life Technologies, Carlsbad, CA, USA).

### 3.3. Histology Study

Formalin-fixed, paraffin-embedded tissue sections of adult human pancreases were examined via double and immunofluorescence staining for the GPR44 antibody (red, Abcam-ab190506, 1:50 dilution), together with major islet hormones, insulin (green, Dako-A0564, 1:200 dilution), glucagon (green, Sigma-G2654, 1:2000 dilution), and somatostatin (green, Dako-A0566, 1:200 dilution). DNA was stained blue with DAPI. Data were verified in human pancreases from three to four independently deceased donors.

### 3.4. General Procedure for the Pretreatment of Precursor JL01-1

#### 3.4.1. Preparation of [K^+^c2.2.2]_2_CO_3_

[K^+^c2.2.2]_2_CO_3_ was prepared using the addition of 1.2 equivalents of Kryptofix 2.2.2 to one equivalent of K_2_CO_3_ in water/MeCN (1:1, *v*/*v*). The solvent was evaporated under reduced pressure and freeze dried.

#### 3.4.2. Preparation of [K^+^c2.2.2]_2_C_2_O_4_

[K^+^c2.2.2]_2_CO_3_ was prepared through the addition of 1.2 equivalents of Kryptofix 2.2.2 to 1 equivalent of K_2_C_2_O_4_ in water/MeCN (1:1, *v*/*v*). The solvent was evaporated under reduced pressure and freeze dried.

#### 3.4.3. Pretreatment of Precursor 4-(*N*-(9-(Carboxymethyl)-2,3,4,9-tetrahydro-1*H*-carbazol-3-yl)-*N*-methylsulfamoyl)-*N*,*N*,*N*-trimethylbenzenaminium

4-(*N*-(9-(carboxymethyl)-2,3,4,9-tetrahydro-1*H*-carbazol-3-yl)-*N*-methylsulfamoyl)-*N*,*N*,*N*-trimethylbenzenaminium (2 mg), K_2_CO_3_ Kryptofix 2.2.2 (5 mg), and K_2_C_2_O_4_ Kryptofix 2.2.2 (12 mg) were dissolved in acetonitrile (1 mL). The resulting mixture was concentrated under reduced pressure. The residue was used in the labeling reaction without further treatment.

### 3.5. Radiosynthesis of [^18^F]TM-30089

#### 3.5.1. Automatic Synthesis of [^18^F]TM-30089

The Synthra RN plus Research module (Synthra GmbH, Hamburg, Germany), a remote-controlled synthesizer, containing one semi-preparative HPLC system, was used for the automatic synthesis of [^18^F]TM-30089.

Typically, for the synthesis of [^18^F]TM-30089, 7.4 GBq (200 mCi) of ^18^F-fluoride (PETNET Solutions Inc., Culver City, CA, USA) was passed through a Sep-Pak Light Waters Accell Plus QMA Cartridge (Waters, Milford, MA, USA). Cartridge-trapped ^18^F-fluoride was eluted into the reactor tube via a solution containing K_2_CO_3_ (20 mg/mL in H_2_O, 0.2 mL) and Kryptofix 2.2.2 (18 mg/mL in CH_3_CN, 0.9 mL). Eluted ^18^F-fluoride was dried via azeotropic distillation, and a solution of pretreated precursor (19 mg) in DMSO (1 mL) was added, then heated at 95 °C for 10 min. After cooling, HPLC buffer (1.8 mL) and 1M hydrochloric acid solution (0.8 mL) were added sequentially. The crude product was purified using a Gemini C-18 (5 µm, 10 × 250 mm) semipreparative column (Phenomenex, Torrance, CA, USA), monitored at 254 nm, and radioactivity via the UV-HPLC/Radio system of the Synthra RNplus module. The mobile phase comprised 45% MeCN in AMF (0.1 M, pH = 4.5), and the flow rate was 4 mL/min. The retention time for the desired product was ~13 min. After concentration under the vacuum for 10 min, the product was formulated in 10% EtOH in saline and was filtered through a 0.22-µM sterile filter. The total synthesis time was ~70 min.

#### 3.5.2. Quality Control (QC)

An Agilent 1260 Infinity II System equipped with a quaternary pump and degasser, an automated sample injector, a column compartment, a variable-wavelength UV detector and a radio detector was used for QC analysis.

QC HPLC conditions: (1) analytical column: Gemini C-18 (5 µm, 4.6 × 250 mm) analytical column (Phenomenex); (2) mobile phase: a gradient of 60% MeCN in water (0.1%TFA) to 85% MeCN in water (0.1%TFA) for a time span of 12 min, at a flow rate of 1.0 mL/min.

The co-injection of the product [^18^F]TM30089 with reference [^19^F]TM-30089 (Chemietek) was confirmed using a RP-HPLC system (retention time was ~6 min).

The overall radiochemical yield of ^18^F-TM30089 was ~20–26% (decay-corrected to EOS) with high radiochemical purity (>99%) and high specific activity (>66 GBq/µmol, decay-corrected to EOS).

### 3.6. Biodistributions

#### 3.6.1. Biodistribution of [^18^F]TM30089 in NOD/SCID Control Mice

The biodistribution of [^18^F]TM30089 was investigated in healthy NOD/SCID mice at various time points post-injection. Three time points (30 min, 60 min, or 90 min post-injection) and one blocking time point (30 min post-injection) were selected in the biodistribution (*n* = 4/each group). “Cold” TM-30089 (1 mg/kg) as the blocking agent was co-injected with [^18^F]TM30089 in the blocking group. Under general anesthesia, NOD/SCID mice were administered doses of tracer (~3.7 MBq) intravenously through the tail vein. At the above-specified time points, the animals were euthanized, and the organs/tissues of interest were harvested. Organs/tissues were weighed, and the gamma was counted. Radioactive uptakes were calculated and reported as percentage of injected dose per gram (% ID/g) for each organ.

#### 3.6.2. Biodistribution of [^18^F]TM30089 in NOD/SCID Mice with 1.1 B4 Cells

The biodistribution of [^18^F]TM30089 was investigated in NOD/SCID mice implanted with 1.1 B4 tumor cells at 30 min post-injection both with and without blocking agent (*n* = 4/each group). “Cold” TM-30089 (1 mg/kg) as the blocking agent was co-injected with [^18^F]TM-30089 in the blocking group. Under general anesthesia, NOD/SCID mice were administered doses of tracer (~3.7 MBq) intravenously through the tail vein, then the animals were euthanized at 30 min post-injection. Organs/tissues/tumors of interest were harvested, weighed, and gamma-counted. Radioactive uptakes were calculated and reported as percentage of injected dose per gram (% ID/g) for each organ/tissue/tumor.

#### 3.6.3. Biodistribution of [^18^F]TM-30089 in NOD/SCID Mice with Transplanted Human Islets in a Renal Capsule

The biodistribution of [^18^F]TM-30089 was investigated in random two NOD/SCID mice with transplanted 500 IEQ human islets (GPR44-enriched) into a renal capsule at 30 min post-injection. Under general anesthesia, NOD/SCID mice were administered doses of tracer (~3.7 MBq) intravenously through the tail vein, then the animals were euthanized at 30 min post-injection. Organs/tissues/islets of interest were harvested, weighed, and gamma-counted. Radioactive uptakes were calculated and reported as percentage of injected dose per gram (% ID/g) for each organ/tissue/islet.

## 4. Conclusions

Coupled with the pretreatment of the unprotected carboxylic acid group of the precursor, a feasible one-step automated synthesis of [^18^F]TM-30089 under mild reaction conditions was successfully achieved. It resulted in a high radiochemical purity (>99%) and a good radiochemical yield (~20–26%), which was able to provide sufficient on-demand radiotracer dosage for pre-clinical and even future clinical uses. This feasible one-step synthesis approach has the potential to significantly propel the progression of ^18^F-labeled GPR44 radiotracer development.

In animal studies, the persistent and specific uptake of [^18^F]TM-30089 was detected in implanted 1.1 B4 cells as well as in renal-subcapsular transplanted human islets, both of which had been shown to exhibit high GPR44 expression. These results suggested that [^18^F]TM-30089 is a promising candidate for targeting GPR44 and further validation is underway. Furthermore, we are currently investigating the mechanism of GPR44 on inflammation-related cancer and diabetes using [^18^F]TM-30089. However, some limitations should be noted. First, the multi-step organic synthesis for this precursor had an impact on the overall chemical yield. Currently, we are focusing on optimizing this synthetic route to improve chemical yield. It is important to note that pigs, non-human primates, and humans exhibit high GPR44 expression, while there is significantly lower or negligible GPR44 expression in mice and rats. When focusing on murine models, the identification of suitable animal models becomes imperative. It is also exciting to observe that more GPR44 murine models have been established and reported.

## Data Availability

Data is contained within the article.
